# Selective prevention programs for substance and behavioral addictions in adolescents: a systematic review

**DOI:** 10.3389/fpsyg.2025.1671822

**Published:** 2025-11-14

**Authors:** Víctor José Villanueva-Blasco, Dalila Eslava, Leticia Olave-Porrúa, José Benito Quintana, Fernando Rodríguez de Fonseca

**Affiliations:** 1 Faculty of Health Sciences, Valencian International University, Castelló de la Plana, Spain; 2 Research Group on Health and Psycho-Social Adjustment (GI-SAPS), Valencian International University, Castelló de la Plana, Spain; 3 Department of Experimental Psychology, Cognitive Processes and Speech Therapy, Faculty of Social Work, Complutense University of Madrid, Madrid, Spain; 4 Aquatic One Health Research Center (ARCUS) & Department of Analytical Chemistry, Nutrition and Food Chemistry, R. Constantino Candeira S/N, Universidade de Santiago de Compostela, Santiago de Compostela, Spain; 5 IBIMA-Plataforma BIONAND, Unidad Clínica de Neurología, Hospital Regional Universitario, Málaga, Spain

**Keywords:** systematic review, selective prevention, addictions, adolescence, evidence, PRISMA

## Abstract

**Justification:**

Selective prevention of substance use among adolescents and young people is a key strategy for reducing risks in vulnerable populations. However, there is a notable lack of systematization and scientific validation of the programs implemented in this field. The aim of this systematic review was to identify existing selective prevention programs, describe their main characteristics, and assess their effectiveness based on the available evidence.

**Method:**

A comprehensive search was conducted in scientific databases (Web of Science, PubMed/MEDLINE, Scopus, and Cochrane Library) and in best practice repositories (Xchange, EDDRA, Portal BBPP Adicciones), following PRISMA criteria and using the MMAT tool for methodological quality assessment. The review was registered in PROSPERO (CRD42024555838).

**Results:**

A total of 20 studies were included, analyzing 24 programs, of which only a portion showed robust evidence according to criteria adapted from the GRADE system. *Preventure, Trampoline, ASSIST*, and *Project TND* stood out for their theoretical foundations, methodological quality, and sustained positive outcomes. Nevertheless, significant limitations were identified: heterogeneity in study designs, limited evaluation in Southern European contexts, and a lack of gender perspective and cultural adaptation.

**Conclusions:**

The findings reveal a gap between practical implementation and empirical research, as many programs lack evaluation of their effectiveness through empirical studies. There is a pressing need to develop rigorously evaluated selective interventions, tailored to sociocultural contexts and aligned with clear quality standards.

## Introduction

For decades, various international organizations have established principles and guidelines for the effective prevention of drug dependence. Among the most prominent are the Center for Substance Abuse Prevention (CSAP) (Cohen et al., 1997; Gardner et al., 2001) and the National Institute on Drug Abuse (NIDA), which has played a key role in promoting the incorporation of scientific evidence into prevention policies (Robertson et al., 2004; Sloboda and David, 1997). In the European context, common quality standards have been promoted, such as the *Minimum European Quality Standards in Drug Demand Reduction* (EQUS; Uchtenhagen and Schaub, 2011), endorsed by the EUDA (formerly the European Monitoring Centre for Drugs and Drug Addiction – European Monitoring Centre for Drugs and Drug Addiction (EMCDDA), 2011, 2013). These principles have been integrated into both European and national strategies, including the EU Drugs Strategy 2021–2025 (Council of the EU, 2021), which emphasizes the need to implement evidence-based prevention programs, apply quality standards, and fund scientifically validated interventions.

The scientific literature distinguishes three levels of prevention (Becoña and Cortés, 2010; Gordon, 1983): universal prevention, aimed at an entire target population regardless of risk or current substance use; selective prevention, aimed at subgroups with a higher risk of substance use; and indicated prevention, aimed at individuals at very high risk who are already using substances or exhibiting behavioral problems.

According to Vázquez et al. (2018), selective and indicated interventions in addictive prevention may focus on: (1) at-risk groups (youth with school absenteeism, behavioral problems, legal offenses, or belonging to vulnerable ethnic minorities); (2) high-risk geographic areas, such as disadvantaged neighborhoods; and (3) families with intergenerational risk, such as those with a history of substance use. Martín (2013) identifies other particularly vulnerable groups, including migrants, individuals with mental health issues, and those facing social and economic exclusion.

Despite the- clear theoretical delineation of these prevention levels and the identification of target groups for selective prevention in addictive prevention, both institutional and scientific attention to these approaches remains insufficient. Compared to the abundant literature on universal programs, research on selective and indicated prevention is still limited (LeNoue and Riggs, 2015). This imbalance is also evident in the scarce presence of selective programs in European best practice repositories such as the Xchange Prevention Registry (European Monitoring Centre for Drugs and Drug Addiction (EMCDDA), n.d.) or the Exchange on Drug Demand Reduction Action (EDDRA), as well as in Spanish platforms such as *Prevención Basada en la Evidencia* (Socidrogalcohol, n.d.) and the *Portal de Buenas Prácticas en Reducción de la Demanda de Drogas y Otras Adicciones* [Portal BBPP Adicciones; Delegación del Gobierno para el Plan Nacional sobre Drogas (DGPNSD), n.d.]. This scarcity makes it difficult for professionals and policymakers to identify and select selective prevention programs with proven efficacy and appropriateness for vulnerable populations.

A recents systematic reviews (Villanueva-Blasco et al., 2024a, 2025) confirmed this trend, showing that the majority of school-based prevention interventions in addictive prevention fall under the category of universal prevention. In contrast, studies on selective and indicated programs remain scarce, are methodologically heterogeneous, and many fail to report significant results or even identify negative effects (Vermeulen-Smit et al., 2015), often limiting their conclusions to preliminary findings (Bröning et al., 2012). This lack of robust evidence compromises the ability to appropriately address the needs of specific at-risk populations.

Although indicated and selective prevention in addictive prevention both target vulnerable populations, this review focuses exclusively on selective prevention for conceptual and methodological reasons. Moreover, indicated prevention studies often rely on clinical samples and less generalizable designs. In contrast, selective prevention allows for earlier intervention, before substance use begins, with greater preventive potential and cost-effectiveness (Gottfredson et al., 2015). This distinction enables a more coherent, useful, and applicable analysis.

Selective prevention targets subpopulations with identified risk factors (biological, psychological, and social) or who live in vulnerable contexts, and it recommends combining actions across family, school, and community settings (United Nations Office on Drugs Crime World Health Organization, 2018; European Union Drugs Agency, 2025). The empirically supported intervention types include: (1) selective family programs focused on positive parenting, communication, and monitoring (United Nations Office on Drugs Crime World Health Organization, 2018; European Union Drugs Agency, 2025); (2) school-based interventions targeting at-risk students that combine life-skills training, decision making, and refusal skills (United Nations Office on Drugs Crime World Health Organization, 2018; European Union Drugs Agency, 2025); (3) brief, targeted modules for impulsivity, sensation seeking, hopelessness, or anxiety sensitivity (Edalati and Conrod, 2019); (4) motivational interviewing and brief interventions, personalized feedback, and coping-skills training (Tanner-Smith and Lipsey, 2015); (5) normative feedback included as a component to correct erroneous perceptions of peer use (Tanner-Smith and Lipsey, 2015); (6) structured alternative leisure and extracurricular activities as part of multicomponent strategies (United Nations Office on Drugs Crime World Health Organization, 2018; European Union Drugs Agency, 2025); and (7) digital formats and group cognitive-behavioral intervention for behavioral addictions (Throuvala et al., 2019).

The literature suggests that many selective interventions in addictive prevention begin in adolescence, when substance use may have already started. Hopson and Steiker (2010) warn that age 15 may be too late to intervene effectively among at-risk youth, emphasizing the need to implement selective prevention at earlier stages. Additionally, it is crucial to ensure that such interventions do not contribute to the stigmatization of participants or exacerbate the existing stigma surrounding drug users, particularly among women. In this regard, the Asociación ADOS (2009) recommends starting with classroom-based group sessions to normalize participation and facilitate the identification of individuals requiring more intensive interventions. According to the EMCDDA guidelines (European Monitoring Centre for Drugs and Drug Addiction (EMCDDA), 2003), selective interventions should be dynamic, participatory, and focused on developing social skills, conflict resolution, support networks, and critical information about substances. These actions should be integrated within a coordinated network involving families, schools, communities, and both social and educational services (Asociación ADOS, 2009; Martín, 2013).

Several systematic reviews have examined selective prevention programs for substance use in the family context (Bröning et al., 2012; Valero et al., 2017; Vermeulen-Smit et al., 2015). While the findings regarding their effectiveness are promising, these reviews consistently highlight the high heterogeneity of the programs, methodological limitations, and the scarcity of evaluations based on randomized controlled trials. Similarly, systematic reviews addressing gambling from a selective prevention perspective have been identified, but none focus on children and adolescents (Grande-Gosende et al., 2020; Monreal-Bartolomé et al., 2023). To our knowledge, no systematic reviews exist that analyze the overall landscape of selective prevention programs for substance and non-substance addictions in adolescents.

Given the urgent need to design interventions adapted to high-vulnerability contexts and the limited body of research on selective programs, conducting a rigorous systematic review is essential. Such a review can guide public policy, funding decisions, and future research toward more equitable prevention strategies for those most in need.

Based on the above, the following research questions were proposed: What selective prevention programs in addictive prevention currently exist targeting populations at risk of substance use? What evidence is available regarding the age of implementation, duration, components, and methodologies used in selective prevention programs? Accordingly, the objectives of this systematic review were: (1) To identify selective prevention programs available in the scientific literature and in European best practice portals, and to determine their main characteristics; and (2) To analyze the effectiveness of these programs and establish recommendation grades based on the available evidence.

## Method

### Search strategy and information sources

To identify existing selective prevention programs, a combined strategy was employed. First, a search was conducted in best practice repositories including Xchange (European Monitoring Centre for Drugs and Drug Addiction (EMCDDA), n.d.), EDDRA (European Monitoring Centre for Drugs and Drug Addiction (EMCDDA), n.d.), *Prevención Basada en la Evidencia* (Socidrogalcohol, n.d.), and the *Portal BBPP Adicciones* (DGPNSD, n.d.). Country and prevention setting filters were applied to identify programs implemented in Spain and within school contexts. Subsequently, a systematic review was carried out following the guidelines of the Preferred Reporting Items for Systematic Reviews and Meta-Analyses (PRISMA) statement (Page et al., 2021). The systematic search was conducted between May 13, 2024, and February 10, 2025. No limits on year of publication were applied; studies from any year were eligible up to the date of the last search (10 February 2025). It was registered in the PROSPERO database (CRD42024555838).

A structured electronic literature search was performed across four databases (Web of Science, PubMed/MEDLINE, Scopus, and Cochrane Library) to retrieve peer-reviewed articles published in English or Spanish, with no restriction on publication date. The search strategy was based on a combination of pre-defined keywords derived from the study objective, following the Population, Intervention, Comparison, and Outcomes (PICO) framework. References retrieved from each database were compiled and managed in a RefWorks library. The searches were conducted and duplicates were removed by the first author of this manuscript.

The search strategy (Table 1) included the keywords: “selective prevention,” “adolescence,” “substance,” “videogames,” “gambling,” “problematic internet use,” “program,” and their synonyms. These terms were combined into the following search string: (“selective prevention” OR “targeted prevention”) AND (“adolescence” OR “adolescents”) AND (“substance” OR “alcohol” OR “tobacco” OR “cannabis” OR “marijuana” OR “drug” OR “gambling” OR “videogames” OR “problematic internet use” OR “internet addiction”) AND (“program” OR “programe”).

**Table 1:** Search strategy implemented on WOS, PubMED, Scopus and Cochrane to conduct the systematic review.

**N°Step**	**Search strategy**
**WOS**	
S1	(((AB=(“selective prevention”)) OR OR (AB=(“targeted prevention”)))
S2	(((AB=(“adolescence”)) OR (AB=(“adolescents”)))
S3	(((AB=(“substante”)) OR (AB=(“alcohol”)) OR (AB=(“tobacco”)) OR (AB=(“marijuana”)) OR (AB=(“drug”)) OR (AB=(“gambling”)) OR (AB=(“videogames”)) OR (AB=(“problematic internet use”)) OR (AB=(“internet addiction”)))
S4	(((AB=(“program”)) OR (AB=(“programe”)))
S5	S1 AND S2 AND S3 AND S4
**PUBMED**	
S1	(“selective prevention”[Title/Abstract] OR “targeted prevention”[Title/Abstract])
S2	(“adolescence”[Title/Abstract] OR “adolescents”[Title/Abstract])
S3	(“substance”[Title/Abstract] OR “alcohol”[Title/Abstract] OR “tobacco”[Title/Abstract] OR “marijuana”[Title/Abstract] OR “drug”[Title/Abstract] OR “gambling”[Title/Abstract] OR “videogames”[Title/Abstract] OR “problematic internet use”[Title/Abstract] OR “internet adicction”[Title/Abstract])
S4	(“program”[Title/Abstract] OR “programe”[Title/Abstract])
S5	S1 AND S2 AND S3 AND S4
**SCOPUS**	
S1	(ABS (“selective prevention”) OR ABS (“targeted prevention”)
S2	(ABS (“adolescence”) OR ABS (“adolescents”)
S3	OR ABS (“substance”) OR ABS (“alcohol”) OR ABS (“tobacco”) OR ABS (“cannabis”) OR ABS (“marijuana”) OR ABS (“drug”) OR ABS (“gambling”) OR ABS (“videogames”) OR ABS (“problematic internet use” OR ABS (“internet addiction”))
S4	(ABS (“program”) OR ABS (“programe”)
S5	S1 AND S2 AND S3 AND S4
**COCHRANE**	
S1	(“selective prevention”):ti,ab,kw OR (“targeted prevention”):ti,ab,kw
S2	(“adolescence”):ti,ab,kw OR (“adolescents”):ti,ab,kw
S3	(“substance”):ti,ab,kw OR (“alcohol”):ti,ab,kw OR (“tobacco”):ti,ab,kw OR (“cannabis”):ti,ab,kw OR (“marijuana”):ti,ab,kw OR (“drug”):ti,ab,kw OR (“gambling”):ti,ab,kw OR (“videogames”):ti,ab,kw OR (“problematic internet use”):ti,ab,kw OR (“internet addiction”):ti,ab,kw
S4	(“program”):ti,ab,kw OR (“programe”):ti,ab,kw
S5	S1 AND S2 AND S3 AND S4

In addition, a backward citation search was conducted by reviewing the reference lists of included studies to identify articles not indexed in the selected databases.

### Eligibility criteria

For the studies identified through the systematic review process, two reviewers (author 2 and author 3) assessed those that met the following inclusion criteria during the initial search stages: (a) inclusion of selective prevention programs; (b) addressing alcohol, tobacco, cannabis use, problematic Internet use, problematic gambling, or video game addiction; (c) targeting adolescent populations; (d) published in peer-reviewed scientific journals; (e) employing randomized controlled trial (RCT) designs; and (f) written in English or Spanish. No limits on year of publication were applied.

The following were excluded: (a) literature reviews, systematic reviews, meta-analyses, books, book chapters, and conference proceedings; (b) studies focused on drug use reduction interventions without published results; (c) non-standardized or non-protocolized preventive interventions; (d) descriptive or quasi-experimental designs; and (e) studies that did not evaluate program outcomes.

### Selection process

**Figure 1:**
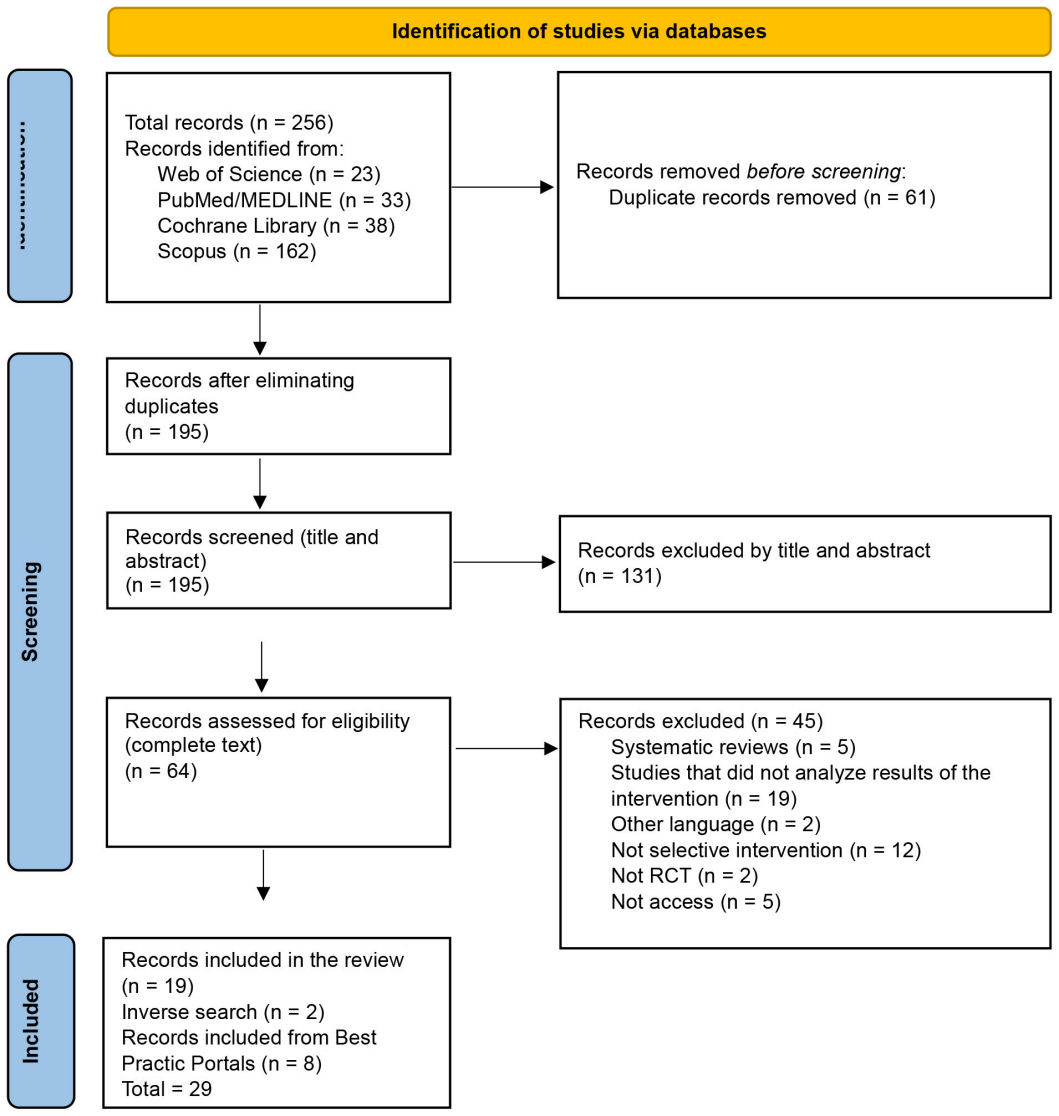
Flowchart.

Two authors (Author 2 and Author 3) conducted study selection in three steps, in accordance with existing literature (Gunnell et al., 2020). First, the titles and abstracts of the articles retrieved from the initial search were screened based on the eligibility criteria. Second, full-text articles were reviewed in detail to assess their eligibility. Third and finally, the reference lists of all included articles were manually reviewed to identify any relevant studies that may have been missed in the initial search strategy. The selection process is summarized in Figure 1, created using the PRISMA flow diagram tool (Haddaway and McGuinness, 2020).

The systematic database review yielded a total of 256 records. After removing duplicates, 195 studies remained for title and abstract screening, of which 131 were excluded. The full texts of 64 articles were reviewed, leading to the exclusion of 45. An additional 2 studies were identified through backward citation searching. Consequently, 21 articles from the database search were included.

Regarding best practice repositories, 4 programs were identified in Xchange, of which 3 were supported by randomized controlled trials (RCTs). Since some of these studies had already been retrieved from the database search, 8 additional studies were reviewed. In the Spanish *Portal de Buenas Prácticas*, one program was found, although it was not supported by any published study. In the *Socidrogalcohol* repository, 9 programs were identified, none of which had evidence from RCTs. The same applied to EDDRA, where 2 programs were located without such evidence.

Therefore, considering the database search and the articles indexed in best practice repositories, a total of 29 studies were included in the final systematic review.

### Data extraction

Three authors (Author 1, Author 2, and Author 3) independently and systematically extracted data from the final list of programs identified in best practice repositories and from efficacy studies retrieved through the systematic review. Multiple categories were identified and presented in separate tables.

First, the characteristics of the selective prevention programs included in this study were summarized, with the following information: (a) program name; (b) country; (c) target population; (d) substance; (e) setting; (f) theoretical model or approach; (g) main components; (h) implementation methodology; (i) number of sessions; and (j) profile of facilitators. Next, the efficacy studies of selective prevention programs were summarized, including: (a) authors; (b) country; (c) study type; (d) sample; (e) substance; (f) intervention name; and (g) outcomes.

Finally, the quality level of the available evidence for each program and its degree of recommendation was presented, including: (a) program name; (b) indexing portal; (c) supporting efficacy studies (authors, year); (d) MMAT score; and (e) recommendation level. Any discrepancies among authors were resolved through consensus. All extracted data were synthesized and organized in tables using Microsoft Excel.

### Methodological quality assessment

The methodological quality of the included articles was assessed using the Mixed Methods Appraisal Tool (MMAT) (Hong et al., 2018). The MMAT is a critical appraisal tool designed for systematic reviews that include quantitative, qualitative, and mixed-method studies. For randomized controlled trials (RCTs), the specific MMAT scale for RCTs was used.

The methodological quality assessment of each study is presented in Table 2. All studies met at least 60% of the MMAT criteria, with an average compliance rate of 81.4%. This high level of methodological quality supports the robustness of the evidence gathered in the review.

**Table 2:** Assessment of methodological quality for RCTs.

**Reference**	**P1**	**P2**	**P3**	**P4**	**P5**	**% de compliance**
Bröning et al. (2019)	Yes	Yes	Yes	Yes	Yes	100%
Campbell et al. (2008)	Yes	Yes	Yes	No	Yes	80%
Champion et al. (2024)	Yes	Yes	No	No	Yes	60%
Conrod et al. (2010)	Yes	Yes	Yes	Yes	Yes	100%
Conrod et al. (2011)	Yes	Yes	Yes	Yes	Yes	100%
Conrod et al. (2013)	Yes	Yes	Yes	Yes	Yes	100%
Debenham et al. (2021)	Yes	Yes	Yes	No	Yes	80%
Geisner et al. (2007)	Yes	Yes	Yes	No	Yes	80%
Goossens et al. (2016)	Yes	Yes	Yes	No	Yes	80%
Kupersmidt et al. (2010)	Yes	Yes	Yes	No	Yes	80%
Lammers et al. (2015)	Yes	Yes	Yes	No	Yes	80%
Lammers et al. (2017)	Yes	Yes	No	No	Yes	60%
Lindenberg et al. (2002)	Yes	Yes	Yes	No	Yes	80%
Lisha et al. (2012)	Yes	Yes	Yes	No	Yes	80%
Lynch et al. (2023)	Yes	Yes	Yes	No	Yes	80%
MacKinnon et al. 1991)	Yes	Yes	Yes	No	Yes	80%
Mahu et al. (2015)	Yes	Yes	Yes	Yes	Yes	100%
Newton et al. (2016)	Yes	Yes	Yes	No	Yes	80%
Newton et al. (2018)	Yes	Yes	No	No	Yes	60%
Newton et al. (2020)	Yes	Yes	Yes	No	Yes	80%
Newton et al. (2022a)	Yes	Yes	Yes	Yes	Yes	100%
Newton et al. (2022b)	Yes	Yes	Yes	Yes	Yes	100%
O'Leary-Barrett et al. (2010)	Yes	No	Yes	No	Yes	60%
Parra-Cardona et al. (2023)	Yes	No	Yes	No	Yes	60%
Perrier-Ménard et al. (2017)	Yes	Yes	Yes	No	Yes	80%
Rodríguez Kuri et al. (2011)	Yes	Yes	Yes	No	Yes	80%
Slade et al. (2021)	Yes	Yes	Yes	Yes	Yes	100%
Starkey et al. (2009)	Yes	No	Yes	No	Yes	60%
Teesson et al. (2017)	Yes	Yes	Yes	No	Yes	80%

P1: Was randomization appropriately performed?; P2: Were the groups comparable at baseline?; P3: Were outcome data complete?; P4: Were outcome assessors blinded to the intervention provided?; P5: Did participants adhere to the assigned intervention?

### Evaluation of the quality of interventions

To determine the quality classification of the analyzed interventions, the MMAT results (Hong et al., 2018) were used. Additionally, an evidence assessment was conducted using the GRADE approach, following the guidelines proposed by Aguayo-Albasini et al. (2014).

Following consensus among the authors, the quality of evidence was classified according to the following criteria: (a) Very low or no evidence, if there was no evidence of effectiveness or if RCTs had a methodological quality score of 40% or lower on the MMAT; (b) Low quality, if some positive effect was found in a study with MMAT quality of 40% or lower; (c) Moderate quality, if a positive effect was found in an RCT with MMAT quality between 60–80%; (d) High quality, if the evaluation was based on an RCT with MMAT quality equal to or greater than 80%.

Regarding the recommendation grades of the interventions, the following criteria were applied: (a) Not recommended, if the evidence quality was low, very low, or nonexistent; (b) Recommended with further studies, if the evidence quality was moderate; (c) Recommended, if the evidence quality was high.

## Results

The included efficacy studies span three continents and six countries: Europe (UK, Netherlands, Germany), North America (USA, Mexico), and Oceania (Australia). Most randomized trials were conducted in the UK and Australia, with additional studies from the USA and the Netherlands; single trials were identified in Germany and Mexico. We did not find eligible RCTs from Africa or Asia. Thus, while the sample is international, evidence is concentrated in Anglo-Saxon and Northern European contexts.

The different outcomes of the systematic review of selective prevention programs for substance and non-substance addictions are presented below.

### Information on the identified selective prevention programs

Table 3 presents information on the selective prevention programs identified. A variety of strategies were observed, ranging from life skills–based approaches (*Aislados, Ludens, Galilei*) to social learning models (*Midwestern Prevention Project, ASSIST*) and culturally adapted programs (*Kamelamos Guinar, CAPAS-Youth*). The implementation contexts vary across school, family, and community settings, with interventions ranging from brief sessions to long-term programs. *Preventure* and *Trampoline* stand out for their strong theoretical foundations and methodological standardization. However, many programs do not clearly specify the number of sessions or provide evaluation outcomes, which limits their replicability and rigorous assessment.

**Table 3:** Characteristics of selective prevention programs.

**Program name**	**Country**	**Target population**	**Substances**	**Setting**	**Model/approach**	**Main components**	**Implementation method**	**Number of sessions**	**Facilitator profile**
Aislados	Spain	11–15 years	Substances	School-based	Life skills approach	Conceptualization, promotion, and generalization of life skills	Role-playing adventure; each session is different	90–180 min per session (number not specified)	Psychologists, community workers, and youth leaders trained in the program
Aprende a Comunicar	Spain	Parents of adolescents and youth (3–18 years)	Substances	Family-based	Multiple theoretical models (social learning, youth reaffirmation, communication theory, CBT, etc.)	Enhancing family communication to strengthen protective factors and perceptions of family dynamics	Activities, exercises, readings, group work	Fifteen sessions	Teachers
ASSIST	UK	12–13 years	Tobacco	School-based	Peer-led learning	Educates on smoking risks and trains peer leaders to promote non-smoking	Peer education, diaries	10 weeks	Health promotion practitioners
CAPAS-Youth	USA	12–14 years, Latino youth with behavioral problems and families	Substances	Family-based	Social interaction learning theory, coercion theory	Culturally and migration-informed promotion of a positive youth environment	Role-playing	Nine sessions	Technicians
Déjame que te cuente algo sobre los porros	Spain	13–16 years	Cannabis	School-based	Health education	Objective, scientific cannabis information addressing myths and risks	Informative and participatory sessions, critical thinking	Variable; generally short sessions aligned with school curriculum	Educators and health professionals trained in prevention
Discosana	Spain	Nightlife workers and young people in nightlife settings	Substances	Community, nightlife	Harm reduction	Objective drug information, self-care, and safe partying resources	Direct interventions, material distribution, training nightlife staff	Variable	Health professionals, social educators, trained volunteers
Drojnet 2	Spain	Youth aged 14–25	Substances	School and community	Prevention and harm reduction	Healthy lifestyles and drug risk awareness	Training, awareness campaigns, participatory workshops, materials	Variable	Health professionals, educators, trained volunteers
Engoe	Spain	Youth, families, at-risk users	Substances	School, family, community	Biopsychosocial perspective, sequential-comprehensive model, multisystemic theory	Healthy lifestyles, responsibility, drug education, youth integration	Not specified	Not specified	Multidisciplinary team (educators, psychologists, social workers)
Galilei	Spain	At-risk adolescents (15–21 years) in vocational programs	Substances	School-based	Inclusive education and life skills	Educational and social reintegration, self-esteem and resilience support	Workshops, tutoring, extracurriculars	Ongoing during the school year	Educators, school counselors, youth workers
Kamelamos Guinar/ Queremos Contar	Spain	Roma adolescents and youth	Substances	Community	Culturally adapted, health promotion and risk prevention	Uses storytelling and oral tradition to address drug use	Participatory workshops with stories and community dialogue	Variable (multi-session cycles)	Cultural mediators, social educators, Roma community members trained in prevention
Rodríguez Kuri et al.	Mexico	At-risk adolescents	Substances	School-based	Theory of planned behavior	Social skills and healthy attitudes, norms and perceptions	Not specified	Five sessions	Psychologists or social workers
The Risk and Resilience Intervention	USA	Women aged 15–24 with low income	Substances and risk behaviors	Community-based	Skills development and motivational learning	Case analysis on drug/alcohol and sexual risks to promote awareness and change	Case studies	Five sessions	Teachers and physicians
The Health Education Intervention	USA	Women aged 15–24 with low income	Substances and risk behaviors	Community-based	Knowledge acquisition to shape attitudes and behaviors	Substance use, pregnancy, and STI brochures and interactive diary	Weekly sessions (5 weeks)	Not specified	
Ludens	Spain	At-risk adolescents (problem gambling)	Gambling	School, community	Life skills and responsible decision-making	Risk awareness, coping, social pressure resistance, healthy leisure	50-min sessions with debates, videos, group dynamics	Two sessions	Prevention technicians (psychologists), counselors
Media Detective	USA	7–13 years	Substances	School-based	Social cognitive theory, dual-process model, theory of reasoned action	Ad deconstruction and active viewer skills	Detective role-play through ad viewing	Ten sessions	Teachers
Midwestern Prevention Project	USA	11–14 years	Substances	School, family, community	Social learning	Expectations, peer/media/adult influence, resistance, social skills	Role-play, feedback, homework	Ten sessions	Trained teachers
Personalized Alcohol Feedback	USA	University students with depressive symptoms	Alcohol	School-based	Social comparison theory	Information on alcohol and depression, normative comparison, moderation tips	Personalized feedback via email	One-time session	Not specified
Preventure	UK	13–16 years with risk-prone personality traits	Substances	School-based	Cognitive-behavioral intervention	Addresses sensation-seeking, impulsivity, anxiety, hopelessness	Interactive group sessions with discussions and role-play	Two 90-min sessions	Psychologists and mental health professionals trained in Preventure
Programa de Competencia Familiar	Spain	At-risk youth aged 7–12 and their families	Substances	Family-based	Risk factor reduction	Emotional/social skills for youth, parenting skills for families	Lectures, discussions, homework	14 sessions each (youth and parents) + 1 joint session	Socio-educational or psychoeducational professionals
Project Toward No Drug Abuse (TND)	USA	At-risk youth aged 14–19 (school failure)	Substances	School-based	Motivation, competence, decision-making model	Substance consequences, perception correction, coping skills	Interactive sessions	Twelve sessions	Educators
Protego	Spain	Parents of children aged 9–13 with behavioral issues	Substances	Family-based	Socio-ecological prevention model	Parenting skills, family bonds, substance stance	Parenting skills sessions	Eight weekly 2-h sessions	Two trained professionals (psychology and prevention)
Rompe Cabezas	Spain	Vulnerable youth aged 16–21	Substances	School, community	Not specified	Risk behavior prevention and life skills promotion	Interactive workshops and group activities	Not specified	Social intervention professionals
Sales Hoy	Spain	Youth aged 15–30 in nightlife spaces	Substances	Community, nightlife	Risk reduction and healthy behavior promotion	Substance use and nightlife risk themes; decision-making tools	Interactive workshops, campaigns, peer reflection	Variable (short-term or long-term programs)	Social educators, youth workers, trained health professionals
Trampoline	Germany	Children of parents with alcohol dependence	Substances	School, community	Psychoeducation and skills-building	Alcoholism education, coping, self-esteem	Support groups with educational/therapeutic activities	Nine weekly 90-min sessions	Psychotherapists and social workers experienced in youth and addictions

The Table 4 summarized the efficacy studies of selective prevention programs, including: (a) authors; (b) country; (c) study type; (d) sample; (e) substance; (f) intervention name; and (g) outcomes.

**Table 4:** Studies evaluating the effectiveness of selective prevention programs.

**Authors (year)**	**Country**	**Study type**	**Sample (N; age range, M age; % female)**	**Substance**	**Intervention name**	**Main findings**
Bröning et al. (2019)	Germany	RCT	*N =* 218 children of substance-using parents (*M =* 9.79, *SD =* 1.87); 47.7% female	Substances	Trampoline	No significant differences between intervention and control groups; both improved from baseline.
Campbell et al. (2008)	UK	RCT	*N =* 5,372 adolescents (12–14 years); 49% female	Tobacco	ASSIST	The likelihood of being a smoker was lower in schools where the intervention was implemented compared to those in the control group immediately after the intervention, as well as one and two years later.
Champion et al. (2024)	Australia	RCT	*N =* 438; *M =* 13.4 (*SD =* 0.47); 63.6% female	Cannabis, stimulants	Preventure	The group that received the Preventure program had significantly lower odds of experiencing cannabis-related harm compared to the control group (OR = 0.78, 95% CI = 0.65–0.92). However, no significant differences were found between the groups in terms of the growth of cannabis use (OR = 0.84, 95% CI = 0.69–1.02) or stimulant use (OR = 1.07, 95% CI = 0.91–1.25) over the 7-year follow-up period.
Conrod et al. (2010)	UK	RCT	*N =* 732; aged 13–16; 64.3% female	Illicit substances	Preventure	The study showed that over a two-year period, the control group increased both the number and frequency of drug use, in contrast to the intervention group. The intervention reduced the likelihood of initiating use of marijuana, cocaine, and other drugs. The effect was strongest in preventing cocaine use.
Conrod et al. (2011)	UK	RCT	*N =* 364; *M =* 14 years; 63.2% female	Alcohol	Preventure	The intervention significantly reduced problematic alcohol use, as well as the frequency of alcohol consumption and binge drinking, compared to the control group. These effects were sustained over the 24-month follow-up period. Among individuals with anxiety symptoms, the intervention also reduced alcohol use for this reason at both 12 and 24 months.
Conrod et al. (2013)	UK	Cluster RCT	*N =* 2643; *M =* 13.7 (*SD =* 0.33); 46.5% female	Alcohol	Preventure	The intervention targeting high-risk (HR) students produced significant effects in reducing alcohol use and binge drinking over a 24-month period, with these students showing a decrease in consumption rates (β = −0.320, *p* =.03) and binge drinking frequency (β = −0.400, *p* =.03). Moreover, low-risk (LR) students also experienced indirect benefits, including reductions in alcohol use rates and in the growth of binge drinking (β = −0.259, *p* =.049; β = −0.244, *p* =.001, respectively).
Debenham et al. (2021)	Australia	Cluster RCT	*N =* 1,005; *M =* 13.4 (*SD =* 0.47)	Tobacco	Preventure	Students who received the intervention reported lower recent tobacco use (OR = 0.66) and reduced intentions to use tobacco (OR = 0.77) compared to controls. The program was particularly beneficial for students with internalizing personality traits, enhancing their self-efficacy to resist peer pressure (OR = 1.85).
Geisner et al. (2007)	USA	RCT	*N =* 177; *M =* 19.28 (*SD =* 1.97); 70% female	Alcohol	Personalized Alcohol Feedback	Although the intervention did not show main effects in reducing alcohol use or alcohol-related problems, students who received feedback on their normative perceptions of alcohol use exhibited significant reductions in their perceived drinking norms compared to the control group. Moreover, those students who successfully reduced their normative perceptions also reported decreases in the total amount of alcohol consumed per week and in alcohol-related problems.
Goossens et al. (2016)	Netherlands	Cluster RCT	*N =* 699; intervention group *M =* 14.0 (*SD =* .98); 53.4% female	Alcohol (binge)	Dutch Preventure Trial	The study found no significant differences in mental health outcomes between the intervention and control groups. At the personality subgroup level, a reduction in anxiety was observed at 12 months among individuals with anxiety sensitivity (AS). However, a negative effect was noted in the negative thinking (NT) group, where depression levels increased in the intervention group compared to the control group.
Kupersmidt et al. (2010)	USA	RCT	*N =* 679; *M =* 9.40 (*SD =* 1.14); 51% female	Alcohol, tobacco	Media Detective	Students in the Media Detective group showed reduced interest in alcohol-related products, lower intentions to use substances among those with prior use, and greater self-efficacy to refuse them. Additionally, they demonstrated improved skills in deconstructing advertisements and a greater understanding of their persuasive intent compared to the control group.
Lammers et al. (2015)	Netherlands	RCT	*N =* 699; *M =* 14.0; 53.4% female	Alcohol	Preventure	Although the program had little to no effect on the overall prevalence of binge drinking, it may have contributed to a reduction in the progression of alcohol use over time.
Lammers et al. (2017)	Netherlands	RCT	*N =* 699; *M =* 14.0; 53.4% female	Alcohol	Preventure	The intervention was effective in reducing alcohol use among adolescents with high levels of anxiety and in decreasing binge drinking among those with high sensation-seeking traits at 12 months post-intervention. Furthermore, adolescents with lower educational attainment showed greater reductions in these behaviors compared to their peers with higher educational levels. However, no significant effects were observed for impulsivity and negative thinking traits, nor were there differences in the program's effectiveness between genders.
Lindenberg et al. (2002)	USA	RCT	*N =* 50 young women; *M =* 19	Substances	Not specified	Both interventions produced consistently similar effects, but neither resulted in a significant reduction in alcohol or cigarette use. However, they led to significant improvements in attitudes, sexual self-efficacy, and resilience levels. Contraceptive use increased among women with partners, and participants also reported an improved ability to communicate with their sexual partners about HIV/AIDS prevention measures.
Lisha et al. (2012)	USA	RCT	*N =* 1426	Drugs	Project TND	The intervention conditions produced effects on the hypothesized mediators, including greater increases in program-related knowledge, greater reductions in drug use intentions, and positive changes in motivation. However, limited generalization was observed regarding attitudes and intentions related to risky sexual behaviors.
Lynch et al. (2023)	Australia	Group RCT	*N =* 701; *M =* 13.44 (*SD =* 0.44); 35.2% female	Alcohol	Preventure	Over a three-year period, a reduction in the growth of alcohol use was observed compared to the control group. However, although the intervention demonstrated significant effects on general psychopathology, no statistically significant effects were found on alcohol use after controlling for general psychopathology. This suggests that improvements in alcohol use may be more closely related to reductions in overall psychopathology than to a direct change in alcohol consumption patterns.
MacKinnon et al. 1991)	USA	RCT	*N =* 5,008	Drugs	Midwestern Prevention Project	The program reduced positive beliefs about drugs and the intention to use them, strengthened the perception that peers were less tolerant of substance use, and improved peer communication about related issues. The change in perceived peer tolerance was the most influential factor in reducing substance use, along with shifts in intentions and beliefs about drug effects.
Mahu et al. (2015)	UK	Group RCT	*N =* 1,038; *M =* 13.7; 44.3% female	Cannabis, alcohol	Preventure	The study findings indicated that brief personality-targeted interventions achieved a significant reduction in marijuana use rates among high-risk adolescents, with a 33% decrease in the likelihood of use at 6 months and reductions in frequency of use at 12 and 18 months. Notably, among the sensation-seeking subgroup, the program demonstrated a marked effect in delaying cannabis initiation, with a significantly lower risk of use (OR = 0.25).
Newton et al. (2016)	Australia	RCT	*N =* 438; *M =* 13.4 (*SD =* 0.47); 18.8% female	Alcohol	Preventure	The intervention group showed a lower rate of alcohol use, a reduction in the prevalence of binge drinking, and decreased alcohol-related harms compared to the control group. Additionally, post-intervention evaluation revealed that 94% of students rated the program as “good” or “very good,” and most reported that the skills acquired would be useful in future situations.
Newton et al. (2018)	Australia	Group RCT	*N =* 1,712; *M =* 13.3 (*SD =* 0.48); 49.5% female	Cannabis	CAP (Climate and Preventure)	Knowledge about cannabis increased among adolescents over a 24-month period. However, no significant differences were found in cannabis use or related harms compared to the control group. There was insufficient evidence to conclude that the interventions were ineffective, and long-term evaluations are recommended.
Newton et al. (2020)	Australia	Group RCT	*N =* 947; *M =* 13.3; 28.8% female	Alcohol	Preventure	The intervention significantly reduced behavioral problems and hyperactivity symptoms compared to treatment as usual (control group).
Newton et al. (2022a)	Australia	Group RCT	*N =* 438; *M =* 13.4 (*SD =* 0.05); 18.8% female	Alcohol	Preventure	The intervention significantly reduced alcohol-related harms over a 7-year follow-up period, showing a decrease in the likelihood of reported harms (OR = 0.81) and a mean reduction in their frequency. However, no significant changes were found in hazardous drinking or binge drinking rates at long-term follow-up. Although the program was effective in reducing binge drinking and hazardous alcohol use at 5.5 years, these effects were not sustained at the 7-year follow-up.
Newton et al. (2022b)	Australia	Group RCT	*N =* 2,190; *M =* 13.3 (*SD =* 0.48); 42.5% female	Alcohol	CAP (Climate and Preventure)	The interventions achieved sustained benefits in alcohol use over a 7-year period, with significantly fewer alcohol-related harms reported in the intervention groups compared to the control group. The Preventure group reported significantly lower weekly alcohol use, and the Climate group showed fewer episodes of heavy drinking. However, the combined group (CAP) did not show a reduction in the risk of harmful alcohol use.
O'Leary-Barrett et al. (2010)	UK	RCT	*N =* 1,159; *M =* 13.7; 43.9% female	Alcohol	Preventure	The intervention group showed a 40% reduction in the risk of alcohol use at six months compared to the control group. Additionally, there was a 55% lower risk of binge drinking among those who were already consuming alcohol at baseline. Participants also reported fewer alcohol-related problems compared to the control group.
Parra-Cardona et al. (2023)	USA (Latino participants)	RCT	*N =* 79; *M =* 13.4; 43.8% female	Alcohol, drugs	CAPAS-Youth	A significant increase in the perceived harm associated with drug use was observed among adolescents, particularly among females. However, no statistically significant changes were found in harm perception among males.
Perrier-Ménard et al. (2017)	UK	Group RCT	*N =* 1,210; *M =* 13.7; 45.5% female	Alcohol	Preventure	The intervention group benefited from the interventions over the 24-month follow-up period, showing improvements in both the total amount of alcohol consumed and the progression of alcohol-related problems.
Rodríguez Kuri et al. (2011)	Mexico	RCT	*N =* 250 (12–15 years); *N =* 96 experimental, *N =* 154 control	Substances	Not named	A statistically significant reduction in the intention to use drugs was observed in the intervention group following participation in the program, whereas the control group exhibited an increase in such intention. Among the antecedent variables of behavioral intention, perceived behavioral control experienced the greatest change. The intervention demonstrated effectiveness under controlled conditions; however, the need to evaluate its applicability in more common real-world settings and among diverse populations is emphasized.
Slade et al. (2021)	Australia	RCT	*N =* 2,190; *M =* 13.3 (*SD =* 0.48); 42.5% female	Alcohol	CAP (Climate and Preventure)	The interventions (Climate, Preventure, and CAP) significantly reduced alcohol use, binge drinking, and alcohol-related harm compared to the control group at the 36-month follow-up. Specifically, the Climate group showed a 74% reduction in the likelihood of alcohol use (OR = 0.26), the Preventure group an 83% reduction (OR = 0.17), and the CAP group a 70% reduction compared to the control group. Additionally, the intervention groups exhibited a smaller increase in binge drinking and a significant decrease in alcohol-related harms.
Starkey et al. (2009)	UK	Cluster RCT	*N =* 10,730; aged 12–13	Tobacco	ASSIST	22% reduction in regular smoking in intervention schools vs. control.
Teesson et al. (2017)	Australia	Group RCT	*N =* 2,190; *M =* 13.3 (*SD =* 0.48); 42.5% female	Alcohol, cannabis	CAP (Climate and Preventure)	Reduced growth in alcohol- and cannabis-related harms in Preventure group.

### Summary of the findings of the included studies

Table 4 summarizes the findings of the included studies, highlighting the effects of various interventions on substance use–related variables. *Preventure* is the most extensively evaluated program, with multiple high-quality studies demonstrating sustained positive effects in reducing alcohol, cannabis, and other drug use—particularly among adolescents with high-risk personality traits. Indirect benefits were also observed among low-risk youth, including improvements in psychosocial skills. *ASSIST* showed efficacy in tobacco use prevention both in the short and long term. In contrast, other programs such as *CAPAS-Youth* or *Personalized Alcohol Feedback* yielded more modest effects, or effects that varied by gender or specific mediating variables. In some studies, significant outcomes were observed only in specific subgroups, or improvements were noted in intermediate variables rather than in actual substance use behaviors.

### Quality of the evidence and recommendation level of the reviewed programs

Table 5 synthesizes the quality of evidence and the recommendation level for the reviewed programs. Only a limited number of programs have sufficient empirical evidence to be strongly recommended, including *ASSIST, The Risk and Resilience Intervention, The Health Education Intervention, Media Detective, Midwestern Prevention Project, Personalized Alcohol Feedback, Project Toward No Drug Abuse (TND), Trampoline*, and the program by Rodríguez Kuri et al. (2011). However, the program that stands out not only for the quality of its evidence but also for the number of supporting studies is *Preventure*. In contrast, many programs implemented in Spain show very low or no levels of empirical evidence, despite being listed in repositories such as *Socidrogalcohol* or the *Portal de Buenas Prácticas (BBPP)*.

**Table 5:** Quality of evidence and recommendation level of selective prevention programs.

**Program**	**Indexing portal**	**Publications supporting effectiveness**	**MMAT score**	**Quality level (recommendation)**
Aislados	EDDRA	—	—	Very low or no evidence (Not recommended)
Aprende a Comunicar	Socidrogalcohol	—	—	Very low or no evidence (Not recommended)
ASSIST	Xchange	Campbell et al. (2008); Starkey et al. (2009)	80%, 60%	High (Recommended)
CAPAS-Youth	Not listed (USA)	Parra-Cardona et al. (2023)	60%	Moderate (Recommended with further studies)
Déjame que te cuente algo sobre los porros	Socidrogalcohol	—	—	Very low or no evidence (Not recommended)
Discosana	Socidrogalcohol	—	—	Very low or no evidence (Not recommended)
Drojnet 2	EDDRA	—	—	Very low or no evidence (Not recommended)
Engoe	Socidrogalcohol	—	—	Very low or no evidence (Not recommended)
Galilei	Socidrogalcohol	—	—	Very low or no evidence (Not recommended)
Kamelamos Guinar/Queremos Contar	Socidrogalcohol	—	—	Very low or no evidence (Not recommended)
Rodríguez Kuri et al.	Not listed (Mexico)	Rodríguez Kuri et al. (2011)	80%	High (Recommended)
The Risk and Resilience Intervention	Not listed (USA)	Lindenberg et al. (2002)	80%	High (Recommended)
The Health Education Intervention	Not listed (USA)	Lindenberg et al. (2002)	80%	High (Recommended)
Ludens	Portal BBPP	—	—	Very low or no evidence (Not recommended)
Media Detective	Not listed (USA)	Kupersmidt et al. (2010)	80%	High (Recommended)
Midwestern Prevention Project	Not listed (USA)	MacKinnon et al. 1991)	80%	High (Recommended)
Personalized Alcohol Feedback	Not listed (USA)	Geisner et al. (2007)	80%	High (Recommended)
Preventure	Xchange	Multiple studies: Champion et al. (2024); Conrod et al. (2010, 2011, 2013); Debenham et al. (2021); Goossens et al. (2016); Lammers et al. (2015, 2017); Lynch et al. (2023); Mahu et al. (2015); O'Leary-Barrett et al. (2010); Perrier-Ménard et al. (2017); Newton et al. (2016, 2018, 2020, 2022a,b); Slade et al. (2021)); Teesson et al. (2017)	Scores range: 60% to 100%	High (Recommended)
Programa de Competencia Familiar	Socidrogalcohol	—	—	Very low or no evidence (Not recommended)
Project Toward No Drug Abuse (TND)	Not listed (USA)	Lisha et al. (2012)	80%	High (Recommended)
Protego	Xchange	—	—	Very low or no evidence (Not recommended)
Rompe Cabezas	Socidrogalcohol	—	—	Very low or no evidence (Not recommended)
Sales Hoy	Socidrogalcohol	—	—	Very low or no evidence (Not recommended)
Trampoline	Xchange	Bröning et al. (2019)	100%	High (Recommended)

## Discussion

The findings of this systematic review provide an updated and critical overview of selective substance use prevention programs targeting adolescents and young people. First, there is a notable underrepresentation of selective programs in best practice repositories, both at the European level (Xchange, EDDRA) and in Spain (Socidrogalcohol, Portal BBPP Adicciones), a gap previously highlighted by other studies (Villanueva-Blasco et al., 2024a). This lack of representation limits professionals' ability to select validated interventions with the potential to be adapted to vulnerable populations.

Despite the general low representation, some programs stand out due to their strong empirical basis and level of recommendation. Among them, *Preventure* emerges as the most scientifically supported program, backed by multiple randomized controlled trials (Champion et al., 2024; Conrod et al., 2010, 2011, 2013; Debenham et al., 2021; Goossens et al., 2016; Lammers et al., 2015, 2017; Lynch et al., 2023; Mahu et al., 2015; O'Leary-Barrett et al., 2010; Perrier-Ménard et al., 2017; Newton et al., 2016, 2018, 2020, 2022a,b; Slade et al., 2021; Teesson et al., 2017). The consistency of its effects, especially among adolescents with high-risk personality profiles, supports the efficacy of personalized interventions. In addition, indirect benefits for low-risk youth and the potential generalization of its effects add further value. In this regard, the evidence underscores the importance of addressing psychosocial vulnerability factors early, in a tailored manner, and based on the identification of individual traits.

In contrast, the majority of programs developed or implemented in Spain present very low or no empirical evidence, despite being included in platforms such as Socidrogalcohol or the Portal de Buenas Prácticas. Some have not even been published in peer-reviewed journals. This disconnect between professional practice and scientific research has previously been noted by authors such as Medina-Martinez and Villanueva-Blasco (2025) and Villanueva-Blasco et al. (2024a, 2025), and highlights the urgent need to establish rigorous evaluation mechanisms for institutionally promoted interventions.

The results also show that most interventions are implemented in school settings, although some are developed in community or family contexts. However, few studies integrate actions from an ecological perspective, despite repeated calls from various organizations (European Monitoring Centre for Drugs and Drug Addiction (EMCDDA), 2003; Asociación ADOS, 2009; Martín, 2013) for family-school-community collaboration to ensure effective prevention for vulnerable youth.

Another relevant finding concerns the age at which interventions are applied. As noted by Hopson and Steiker (2010), initiating prevention at age 15 may be too late in high-risk contexts. However, the results show that few interventions target earlier ages or incorporate developmentally staged strategies.

Regarding program components, those that integrate the development of social skills, emotional coping, and decision-making (such as *Preventure, Trampoline*, or *Project TND*) show more favorable outcomes compared to those focusing solely on substance-related information.

Regarding the limitations of the studies included in this systematic review, methodological heterogeneity represents a significant constraint. While many studies meet minimum quality standards, others show limitations related to assessor blinding, adherence to the intervention, or the absence of long-term follow-up. These issues hinder the comparability of findings and the generalizability of conclusions. Additionally, some programs exhibit gender-specific effects (e.g., *CAPAS-Youth*), highlighting the need to incorporate a gender perspective in the design and evaluation of interventions.

Finally, this review also reveals a lack of systematic evaluation of programs targeting specific populations such as migrant youth, ethnic minorities, or adolescents at social risk—despite these groups being repeatedly identified as priority populations (Martín, 2013; Vázquez et al., 2018). The scarcity of culturally adapted interventions, such as *Kamelamos Guinar*, limits equity in prevention efforts and exacerbates existing disparities.

### Preventive implications and public policy recommendations

The findings of this systematic review have important implications for both preventive practice and the design and implementation of public policies in the field of addictions. Firstly, the limited presence of validated selective prevention programs in European and Spanish best practice repositories highlights the need to strengthen the systematic evaluation of currently implemented interventions. Many locally deployed programs lack sufficient empirical support, making it difficult to ensure their effectiveness, efficiency, and appropriateness for the characteristics of target populations.

In this regard, public administrations should prioritize funding for preventive programs with strong scientific backing, as recommended by both European and national drug strategies (Council of the EU, 2021; Delegación del Gobierno para el Plan Nacional Sobre Drogas (DGPNSD), 2018) and by studies such as Villanueva-Blasco et al. (2024b). Moreover, it is essential to establish mechanisms that condition the inclusion of programs in official registries on meeting minimum scientific evidence criteria, thus preventing the dissemination of interventions without rigorous evaluation (Medina-Martinez and Villanueva-Blasco, 2025).

Programs that have demonstrated effectiveness, such as *Preventure, ASSIST*, and *Trampoline*, are characterized by a clear theoretical foundation, structured components, and adaptability to different risk profiles. This suggests that preventive policies should focus on implementing personalized models based on specific vulnerability factors, such as personality traits, family context, or membership in minority groups.

Furthermore, the findings show that the most effective interventions integrate psychoeducational content, training in social and emotional skills, decision-making, and stress management, going beyond the mere transmission of information. Therefore, public policies should support the training of professionals in active and participatory methodologies and promote coordination among the educational, health, and social care systems in order to provide integrated responses.

Finally, there is limited attention to highly vulnerable populations, such as youth with school attendance issues or academic failure, those with symptoms indicative of mental health problems, migrants, ethnic minorities, or adolescents from dysfunctional family environments. In this context, policies should promote the development and validation of culturally sensitive interventions that incorporate a gender perspective and are focused on health equity, with the aim of reducing disparities and increasing preventive impact. In such cases, coordinated and complementary preventive work with the mental health system, primary care, and social services network is strongly recommended. This coordination should include proper monitoring of the progression of addictive behaviors and, when necessary, the implementation of indicated prevention strategies.

## Limitations

This systematic review presents several limitations that should be considered when interpreting its findings. First, the considerable methodological heterogeneity among the included studies (in terms of design, settings, populations, and program characteristics) hinders direct comparisons and limits the feasibility of conducting a meta-analysis. Additionally, although a comprehensive search was carried out, it is likely that some locally implemented selective programs are neither published nor indexed in official databases or repositories, introducing a potential availability bias.

Some studies do not clearly report key elements. The lack of analysis of moderating variables such as gender, ethnicity, or socioeconomic status reduces our understanding of for whom and under what conditions the interventions are most effective—an issue particularly relevant when addressing vulnerable populations. Future research should address this gap by incorporating variables such as gender, sexual orientation, cultural background, and ethnicity, which may influence program effectiveness and, therefore, suggest the need for culturally and contextually adapted preventive interventions.

Moreover, most studies originate from Anglo-Saxon or Northern European contexts, raising questions about the transferability of findings to other sociocultural realities, where risk dynamics and preventive systems may differ substantially. This geographic concentration also raises important questions regarding the representativeness of the knowledge generated in the field of prevention. Why have more intervention programs been identified in these countries? One possible explanation is that Anglo-Saxon and Northern European contexts have a stronger tradition of systematically evaluating interventions, which translates into a higher volume of scientific output and greater availability of studies meeting the quality standards required by systematic reviews. This disparity does not necessarily imply the absence of programs in other regions, but rather a potential lack of systematization, documentation, or dissemination of such initiatives in the scientific literature. Therefore, it is crucial to expand the geographical scope of research by promoting the rigorous evaluation of programs in diverse sociocultural contexts (particularly in regions such as Latin America, Asia, Africa, and Southern Europe) in order to enrich the global evidence base and enhance the applicability of the findings. These limitations reinforce the need for continued research using rigorous designs, improved documentation, and greater sensitivity to contextual and population diversity in selective addiction prevention. It is essential to evaluate the effectiveness of programs in underrepresented contexts, as well as to explore how various cultural, social, and structural variables influence the implementation and outcomes of interventions.

Beyond the limitations identified, it is also important to incorporate complementary tools that enhance the targeting of selective interventions and enable their effectiveness to be evaluated. In this regard, wastewater analysis has emerged as a reliable method for detecting real patterns of substance use in specific geographic areas and is well-established for large populations (e.g., Bijlsma et al., 2021; European Union Drugs Agency, 2025; González-Mariño et al., 2020). Its use allows for the identification of high-risk geographic areas and can guide the implementation of prevention programs where they are most needed. This strategy could improve the territorial alignment of actions, contributing to more effective and population-sensitive planning.

In addition, recent studies have demonstrated the utility of this methodology on a smaller scale, such as in correctional institutions (Egaña et al., 2025), and to a lesser extent in secondary education settings (Verovšek et al., 2021, 2023). While wastewater analysis in these contexts poses ethical and methodological challenges, when conducted with careful planning, anonymity, and confidentiality, it may serve as an effective tool for identifying key threats and evaluating the impact of prevention programs.

## Conclusions

This systematic review provides a critical perspective on the current state of selective prevention of substance and behavioral addictions among adolescents and young people, highlighting both advances and persistent gaps in the field. Although several programs with high levels of empirical evidence were identified, such as *Preventure, Trampoline, ASSIST*, and *Project TND*, the majority of selective interventions lack rigorous scientific validation. This disconnect between practical implementation and empirical evaluation represents a significant challenge for the development of effective, evidence-based public policies.

The findings show that the most effective programs share common characteristics: a clear theoretical foundation, personalization based on risk factors, and the use of participatory methodologies focused on developing psychosocial skills. However, important limitations were also identified, particularly regarding the generalizability of results due to methodological heterogeneity and the lack of attention to key variables such as gender, sociocultural context, or age of onset.

Overall, this review underscores the need to promote more rigorous and culturally adapted research, as well as to strengthen quality standards in the selection of prevention programs that are publicly funded and implemented. Advancing a professionalized, evidence-based, and equity-focused approach to selective prevention is essential to ensuring greater health equity and more effective responses for the most vulnerable populations.

## Data Availability

The raw data supporting the conclusions of this article will be made available by the authors, without undue reservation.
